# Advances in the Mechanism of ceRNA Regulation in Postoperative Cognitive Dysfunction

**DOI:** 10.2174/011570159X391415250707115740

**Published:** 2025-09-11

**Authors:** Qiang Liu, Lin-Hui Ma, Chen-Rui Zhou, Tian-Qi Chen, Wei-Feng Wu, Hui-Hui Miao, Yu-Qing Wu, Cheng-Hua Zhou

**Affiliations:** 1 Department of Anesthesiology and Perioperative Medicine, Shanghai Key Laboratory of Anesthesiology and Brain Functional Modulation, Clinical Research Center for Anesthesiology and Perioperative Medicine, Translational Research Institute of Brain and Brain-Like Intelligence, Shanghai Fourth People's Hospital, School of Medicine, Tongji University, Shanghai, 200434, China;; 2 Department of Anesthesiology, National Cancer Center/National Clinical Research Center for Cancer/Cancer Hospital, Chinese Academy of Medical Sciences and Peking Union Medical College, Beijing, 100021, China;; 3 Jiangsu Province Key Laboratory of Anesthesiology, NMPA Key Laboratory for Research and Evaluation of Narcotic and Psychotropic Drugs, Xuzhou Medical University, Xuzhou, 221004, China;; 4 Department of Anesthesiology, Beijing Shijitan Hospital, Capital Medical University, Beijing, 100038, China;; 5 Jiangsu Key Laboratory of New Drug Research and Clinical Pharmacy, Xuzhou Medical University, Xuzhou, 221004, China

**Keywords:** Postoperative cognitive dysfunction, competing endogenous RNAs, microRNA, long non-coding RNA, circular RNA, central nervous system

## Abstract

Postoperative cognitive dysfunction (POCD) is a common central nervous system complication in elderly patients after surgery, characterized by cognitive changes, including impaired learning and memory, reduced attention, and mental disorders and personality changes in severe cases. Despite extensive research, effective targeted therapies remain elusive, underscoring the urgent need to elucidate their molecular mechanisms and identify novel therapeutic targets. Non-coding RNAs (ncRNAs), major transcription products of the human genome, are highly expressed in the central nervous system and play critical roles in regulating neuronal and synaptic complexity through interactions with other biomolecules. Notably, certain ncRNAs modulate gene expression networks by regulating miRNAs, a phenomenon known as the competing endogenous RNA (ceRNA) mechanism. In this review, we summarized and analyzed emerging evidence on ceRNA-mediated regulatory mechanisms in POCD pathogenesis, aiming to establish a foundation for future mechanistic exploration and therapeutic development.

## 1 INTRODUCTION

POCD has been studied for more than half a century since Bedford first proposed the clinical phenomenon of cognitive decline in patients after anesthetic surgery in 1955 [1]. POCD remains a common neurological complication in elderly patients after surgery. Research indicates that approximately 40% of elderly surgical patients over the age of 60 experience POCD at the time of discharge, and about 10% continue to suffer from POCD three months later [2-4]. Clinically, POCD is associated with prolonged hospitalization, diminished quality of life, increased postoperative complications, and higher mortality rate, imposing substantial socioeconomic burdens [5, 6]. The current understanding of POCD pathogenesis implicates multiple mechanisms, including oxidative stress, neuronal apoptosis, neuroinflammation, the apolipoprotein E4 (ApoE4) polymorphism, and amyloid-β (Aβ) deposition, as well as tau protein hyperphosphorylation [7-12]. While these findings have advanced our knowledge of POCD, they remain insufficient for developing effective therapeutic strategies. Recently, attention has been gradually increasing toward the role of non-coding RNA (ncRNA) in POCD [13, 14]. Several evidence indicate that ncRNAs participate in the pathogenesis and progression of the disease by regulating gene expression, protein translation, synaptic plasticity, and neuroinflammation [15-19]. In the future, ncRNA-based molecular diagnostics and targeted therapies may offer novel strategies for preventing and treating POCD.

ncRNAs represent a diverse class of RNA molecules that lack protein-coding capacity yet play pivotal regulatory roles in cellular physiology and pathogenesis. These functional RNAs exert their biological effects through direct modulation of gene expression programs and dynamic interactions with various biomolecules [20]. Research has shown that lower organisms, such as Caenorhabditis elegans, have a comparable number of protein-coding genes to humans. However, the human genome is ~30 times larger than that of *Caenorhabditis elegans*, suggesting that the non-coding portion of the genome is critical in determining the functional complexity of higher eukaryotes [21]. Indeed, approximately 98% of the human genome is transcribed into non-coding transcripts, while only about 2% of transcripts encode proteins [22, 23]. The central nervous system (CNS) exhibits particularly abundant and diverse ncRNA expression profiles compared to peripheral tissues [24] with distinct spatiotemporal expression patterns that are evolutionarily conserved [25, 26], This remarkable tissue specificity and developmental regulation underscore ncRNAs' crucial involvement in neural circuit formation, synaptic plasticity, and higher cognitive functions. Mounting evidence has established the pathogenic contributions of dysregulated ncRNAs in various neurological disorders, including postoperative cognitive dysfunction [27-29], Parkinson’s disease [30-32], traumatic brain injury [33, 34], and Alzheimer’s disease [35-38]. Based on molecular size, ncRNAs are broadly categorized into two classes: small ncRNAs (<200 nucleotides; *e.g*., microRNAs, piRNAs) and long ncRNAs (>200 nucleotides; *e.g*., lncRNAs, circRNAs). This review provides a comprehensive summarization of ncRNA classification, molecular signatures, and their emerging roles in CNS pathophysiology.

We conducted a comprehensive literature search in the PubMed database for English-language publications. The primary search terms included: postoperative cognitive dysfunction or perioperative neurocognitive disorders, in combination with ceRNA, lncRNA, circRNA, piRNA, eRNA, and miRNA. Given the emerging nature of this research field, no restrictions were applied to publication dates. Through manual screening of the retrieved articles, we selected the most relevant studies based on rigorous evaluation of their quality and scientific relevance.

## 2 CLASSIFICATION AND MOLECULAR CHARACTERISTICS OF ncRNAs

### 2.1 miRNAs

MicroRNAs are the most abundant non-coding RNAs in the central nervous system, with approximately 70% of identified and characterized miRNAs expressed in the brain. Furthermore, half of those miRNAs with tissue-specific expression patterns are brain-specific [39]. Mature miRNAs are about 18-25 nucleotides in length. Typically, miRNAs can complementarily pair with bases in the 3'-untranslated region (UTR) of target mRNAs to form RNA-induced silencing complexes (RISCs), which lead to the translational repression or degradation of mRNAs, thereby regulating gene expression at the post-transcriptional level [40]. In planta, miRNAs are usually perfectly complementary to the bases of target mRNAs, thus leading to mRNA degradation. In contrast, in most animals, the interaction between miRNAs and target mRNAs requires only partial base complementation, with the 2^nd^-7^th^ nucleotides, known as the seed region, being most critical for target recognition. Consequently, one miRNA can bind to multiple mRNAs, with studies predicting that 50% of miRNAs can bind to 1-400 mRNAs, and some to as many as 1,000 [41]. The diversity of miRNA species, along with their functional complexity, determines the range of their biological functions. It has been predicted that more than one-third of human genes are regulated by miRNAs [42]. miRNAs play important roles in learning, memory, neurological development, and neurological diseases [43]. Additionally, miRNAs can cross the blood-brain barrier and are easily detected. The expression profiles of miRNAs can serve as indicators for predicting and diagnosing neurological diseases. Future multicenter studies will likely enhance the potential of miRNAs as reliable biomarkers, and further research will continue to uncover more of their biological functions.

### 2.2 LncRNAs

Long non-coding RNAs (lncRNAs) are a class of non-coding RNAs with the longest sequences, containing more than 200 nucleotides [44]. LncRNAs are abundant and conserved in the brain [45, 46], and it is known that there are approximately 96,308 genes encoding 172,216 unique lncRNA transcripts in the human genome [47].

LncRNAs are similar to mRNAs in terms of sequence length and expression characteristics; however, unlike mRNAs, lncRNAs do not function as proteins. Studies have shown that lncRNAs can act as scaffolding molecules (especially on chromatin), sponges (binding to miRNAs or other RNAs), recruitment molecules, splicing regulators, or by binding to proteins [48-50] to regulate numerous physiopathological processes [51-54]. Recent studies have shown that lncRNAs function through two main mechanisms: cis-acting, which influences the expression of neighboring genes, and trans-acting, where lncRNAs control the expression, transcription, or other cellular functions of genes far from their specific transcription site [44]. Although lncRNAs do not have protein-coding functions, their spatial and temporal-specific expression patterns suggest that lncRNAs are potential biomarkers and therapeutic targets for clinical diagnosis and treatment. For example, the knockdown of lncRNA SNHG1 in rat blood can inhibit the inflammatory response, slowing the progression of osteoarthritis and reducing pain [55]. Additionally, lncRNA TLNC1 promotes the growth and metastasis of hepatocellular carcinoma by inhibiting p53 signaling [56]. In addition, lncRNAs have been shown in various neurodegenerative diseases such as Alzheimer's disease (AD), Parkinson's disease (PD), multiple sclerosis (MS), Huntington's disease (HD), and schizophrenia [57-59]. Further in-depth functional experiments are needed to reveal the roles of lncRNAs in disease regulation in the future.

### 2.3 circRNAs

Circular RNAs (circRNAs) are a special class of non-coding RNAs that, unlike linear RNAs, have a circular structure. They are generated by back-splicing the exons of precursor mRNAs and, therefore, do not contain the N7-methylguanosine (m7G) cap at the 5’ end or the polyadenylate tail at the 3’ end. CircRNAs have been discovered relatively recently, and studies over the last decade have shown that they are widely expressed in biological systems [60-63]. More than 183,000 circRNAs have been identified from human gene transcripts, over 96,000 from macaque transcripts, and more than 82,000 from mouse transcripts [64, 65]. Although circRNAs are typically expressed at low levels, there is evidence that circRNAs are significantly enriched in the mammalian brain relative to other tissues [63, 66]. Interestingly, You *et al.* found that a disproportionate number of circRNAs originate from host genes encoding synaptic proteins and that circRNAs are more abundant at synaptic sites than their linear isoforms [67]. This differential expression pattern is exhibited across tissues, cell types, and cell compartments, suggesting the great potential of circRNAs in regulating biological functions.

Previous studies have demonstrated that circRNAs are involved in cell proliferation and tumorigenesis [68-70], immune responses [71-74], and the regulation of neuronal function [67, 75]. Misregulation of circRNAs has been associated with the development of human diseases, particularly neurodegenerative disorders. For example, a study on circRNAs in Alzheimer’s disease (AD) and related dementias identified 48 circRNAs significantly associated with AD, with circRNA expression varying by dementia subtype [76]. A genome-wide circRNA expression profiling of postmortem brains of autistic patients found that 60 circRNAs were aberrantly expressed in autistic patients [77]. In addition, circRNAs have been associated with the development of Parkinson's disease (PD), traumatic brain injury (TBI), stroke, and postoperative neurocognitive deficit (PND) [30, 78]. Given the stable structure of circRNAs and their variability in temporal and spatial expression, further investigation is necessary to explore their potential as biomarkers and therapeutic targets.

### 2.4 piRNAs

piRNAs (Piwi-interacting RNAs) represent a unique class of small non-coding RNAs, typically 21-35 nucleotides in length, that specifically interact with Piwi proteins of the Argonaute family [79]. These molecules are highly abundant in animal germline cells and play crucial roles in maintaining genomic stability and regulating reproductive development. Current genomic analyses have identified tens of thousands of unique piRNA sequences across species, with their expression being particularly enriched in gonadal tissues [80]. Recent studies have demonstrated their involvement in somatic gene regulation, antiviral defense, and even tumorigenesis [81, 82]. For instance, aberrant piRNA expression has been associated with testicular germ cell tumors and infertility in both humans and model organisms [83, 84]. Furthermore, emerging research suggests potential roles for piRNAs in neurological disorders, with altered piRNA profiles observed in models of Alzheimer's disease and Parkinson's disease [85-87]. The tissue-specific expression patterns and functional diversity of piRNAs underscore their potential as diagnostic biomarkers and therapeutic targets, particularly for reproductive disorders and certain types of cancer. However, no studies on piRNAs have been reported in POCD models to date, and this area remains to be fully elucidated.

### 2.5 eRNAs

Enhancer RNAs (eRNAs) represent a class of non-coding RNAs transcribed from genomic enhancer regions, typically ranging in length from 200 to 1000 nucleotides [88, 89]. Unlike lncRNAs, eRNAs exhibit extremely short half-lives (ranging from minutes to hours) and serve as direct molecular indicators of enhancer activity. Current genomic studies estimate that tens of thousands of enhancer regions actively transcribe eRNAs across different cell types, with their expression demonstrating high cell-type specificity and strong correlation with target gene activation [90].

Existing evidence suggests that aberrant eRNA expression is linked to various disease states. In cancer, dysregulated eRNAs have been implicated in the activation of oncogenes [91, 92]. In neurological disorders, altered eRNA profiles have been observed in Alzheimer's disease models, where they may contribute to synaptic dysfunction [93]. The highly cell-specific nature of eRNA expression makes them attractive candidates as diagnostic biomarkers, although their inherent instability presents technical challenges for clinical applications.

## 3 THE ROLE OF THE ceRNA MECHANISM IN POCD

A pivotal advancement in non-coding RNA (ncRNA) biology emerged in 2011 when Pandolfi's team proposed the competing endogenous RNA (ceRNA) hypothesis, which revolutionized our understanding of ncRNA-mediated gene regulation [94]. This paradigm-shifting theory demonstrated that ncRNAs not only function autonomously but also participate in sophisticated regulatory networks through molecular crosstalk. The core mechanism involves miRNAs serving as central hubs that recognize specific binding sites (miRNA response elements, MREs) on various RNA molecules and mediate mRNA inhibition or degradation through the formation of miRNA-RISC complexes. Remarkably, ceRNAs (including lncRNAs, circRNAs, and pseudogenes) can compete for shared MREs, thereby sequestering miRNAs and derepressing their target mRNAs. This regulatory architecture exhibits extraordinary complexity, as individual miRNAs can regulate hundreds of transcripts, while most ceRNAs contain multiple MREs, collectively forming an extensive and interconnected regulatory network.

While the ceRNA hypothesis was initially characterized in oncological contexts, emerging evidence has revealed its significant implications for POCD pathogenesis. This review systematically categorizes and summarizes different types of ceRNAs involved in POCD regulation.

### 3.1 Role of lncRNA-miRNA-mRNA Regulatory Network in POCD

**Fig. (1):**
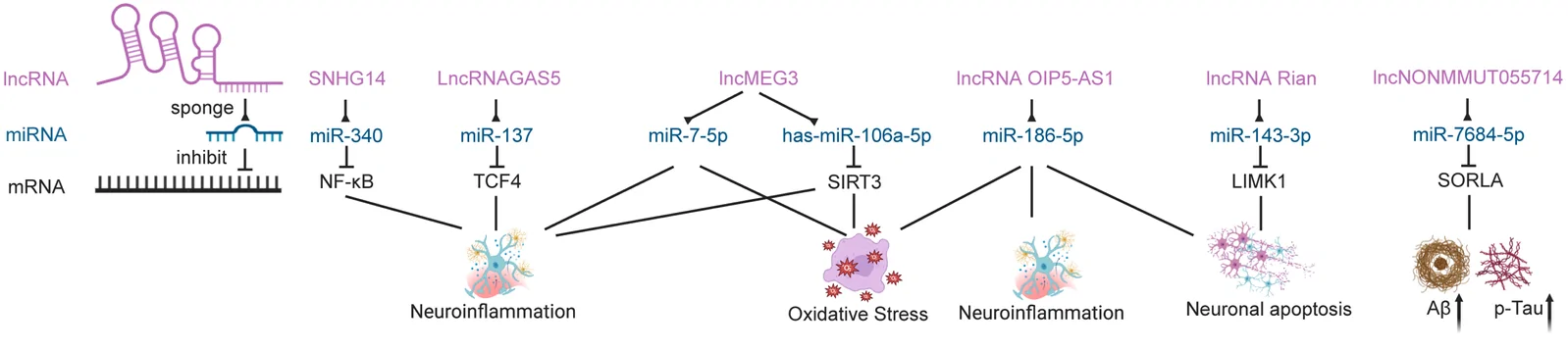
Role of lncRNA-miRNA-mRNA regulatory network in POCD. LncRNAs sponge with miRNAs which inhibit the target mRNA. The lncRNAs are colored in purple, miRNAs are colored in blue, and mRNAs are colored in black.

Recent studies have revealed that long non-coding RNAs (lncRNAs) represent a new class of RNAs with significant biological functions. LncRNAs have been shown to regulate protein expression through the competitive endogenous RNA (ceRNA) mechanism. By competitively binding to miRNAs through MREs, lncRNAs can derepress miRNAs from their target gene mRNAs, thereby upregulating the relevant protein levels. The involvement of lncRNAs has been previously found in several models of cognitive dysfunction caused by inhalation anesthetics [95, 96], and the role of lncRNAs as ceRNAs in POCD has been validated (Fig. **1**).

The aging brain shows significant vulnerability to stress, and excessive inflammation in the brain after anesthetic surgery in elderly patients is the key mechanism in the development of POCD. Several studies have demonstrated that lncRNAs can act as active molecules involved in POCD pathogenesis by regulating CNS inflammation. Zhang *et al*. found that lncRNA GAS5 was upregulated in a POCD model, inducing neuroinflammation and cognitive decline by inhibiting miR-137, which upregulates TCF4 expression [97]. Wei *et al.* showed that silencing of lncNONMMUT055714 in POCD model mice led to increased Aβ and p-tau expression, elevated inflammatory markers, activation of oxidative stress, and neuronal apoptosis *in vitro* through the upregulation of miR-7684-5p and inhibition of SORLA expression. Conversely, overexpression of lncNONMMUT055714 prevented cognitive decline in the POCD model mice [98]. Similarly, lncRNA Rain is involved in sevoflurane-induced cognitive dysfunction by regulating the expression level of Lim kinase 1 (LIMK1) through miR-143-3p [99], and lncRNA OIP5-AS1 exerts a protective effect on isoflurane-mediated POCD by targeting miR-186-5p [100].

Furthermore, in an isoflurane-induced POCD model, lncMEG3 enhances neuroinflammation and oxidative stress in rats by directly sponging miR-7-5p, leading to increased neuronal apoptosis [101]. Notably, lncMEG3 demonstrated an opposite function in another study, where it inhibits inflammatory responses and oxidative stress *via* the miR-106a-5p/SIRT3 axis, thereby alleviating POCD [102]. The conflicting results of these two studies are thought-provoking. One key factor may lie in the differences between the animal models. Li *et al*., [101] used a rat model to investigate isoflurane-induced cognitive dysfunction, whereas Ye *et al.* [102] employed a mouse model undergoing orthopedic surgery under sevoflurane anesthesia. Regarding mechanistic investigations, both studies performed similar molecular experiments but in different cell types: HT22 hippocampal neuron cells and BV-2 mouse microglial cells, introducing a potential confounding variable. These methodological differences underscore the need to establish standardized protocols for POCD modeling to enable more reliable comparisons across studies. However, even if model discrepancies do not fully account for the opposing findings, the role of lncMEG3 in POCD warrants closer scrutiny. The expression level or subcellular localization of lncMEG3 may differ in neurons *versus* microglia. Apoptosis may be promoted through miR-7-5p in neurons, whereas inflammation is inhibited through miR-106a-5p in glial cells. Furthermore, existing evidence suggests that lncMEG3 may interact with multiple miRNAs, competing for binding and thereby modulating inflammation through various downstream pathways. This complexity reflects the nature of ceRNA networks, which function as intricate regulatory systems rather than straightforward linear cascades. The development of bioinformatics technology has revealed this feature well. Recently, Wei *et al*. successfully used bioinformatics methods to construct lncRNA-miRNA-mRNA networks, highlighting their roles in POCD. This study identified 175 lncRNAs, 117 mRNAs, and 26 miRNAs that were differentially expressed in the hippocampus of POCD model mice *via* microarray analysis. The ceRNA network constructed by bioinformatics suggested that lncRNA NONMMUT055714 competitively binds to miR-7684-5p, thereby increasing the expression of the target gene Sorl1 [103]. These potential regulatory networks provide future research directions and potential intervention targets for POCD. Recent studies have shown that dexmedetomidine alleviates neuroinflammation and cognitive dysfunction after anesthetic surgery in aged mice *via* the SNHG14/miR-340/NF-κB axis [104]. The complexity of the regulatory network and the potential for protection methods suggest that further investigation of lncRNAs and their associated ceRNA mechanisms in POCD is warranted.

### 3.2 Role of circRNA-miRNA-mRNA Regulatory Network in POCD

**Fig. (2):**
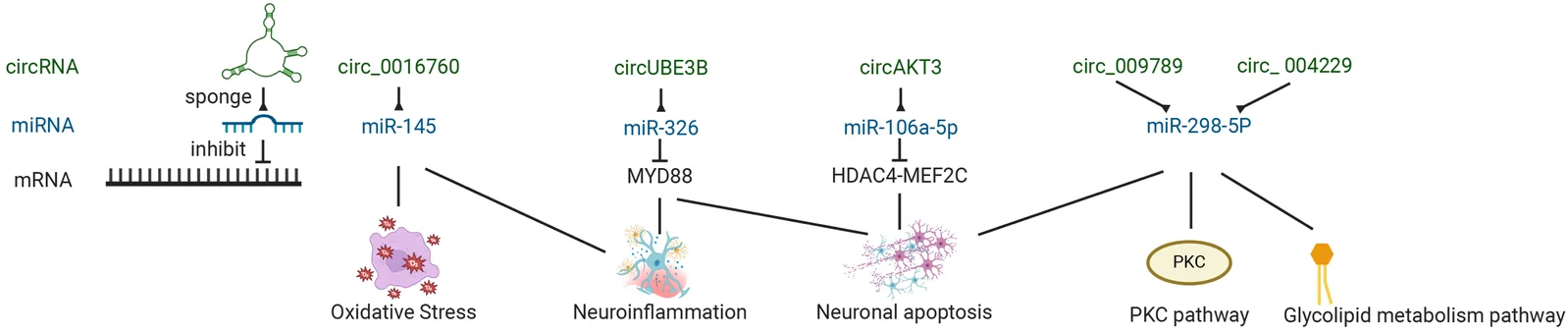
Role of circRNA-miRNA-mRNA regulatory network in POCD. CircRNAs sponge with miRNAs which inhibit the target mRNA. The circRNAs are colored in green, miRNAs are colored in blue, and mRNAs are colored in black.

LncRNAs are not the only elements in the ceRNA network that regulate the pathogenesis of POCD; it has also been found that circRNAs are involved (Fig. **2**). Recently, it was found that circAKT3 is associated with hippocampal neuronal apoptosis in a POCD model mouse. Interestingly, circAKT3 was found to be involved in regulating neuronal apoptosis through modulation of the miR-106a-5p/HDAC4/ MEF2C signaling pathway, as determined by bioinformatics analysis, as well as *in vitro* and *in vivo* experiments, highlighting the role of circRNA-miRNA and miRNA-mRNA interactions in the ceRNA regulatory network related to POCD [13]. In addition, recent studies demonstrated that circUBE3B dysregulation mediated sevoflurane-induced hippocampal nerve injury and POCD by targeting the miR-326/MYD88 network [105]. Furthermore, circ_0016760 was found to regulate sevoflurane anesthesia-induced cognitive behaviors, inflammatory cytokines, and oxidative stress abnormalities by targeting miR-145 [106].

Apart from the above experimentally verified ceRNA network associated with POCD, other circRNA-associated ceRNA networks (cirCeNETs) have been identified primarily through transcriptome mapping studies in various animal models and patient samples. Through high-throughput transcriptome sequencing, researchers constructed the cirCeNET in a mouse model of POCD and performed functional enrichment analyses to identify potential aberrant mechanisms. An enrichment analysis of relevant transcriptomes in the hippocampal region of aged POCD mice suggested that the circRNAs-associated ceRNA network may be involved in POCD pathogenesis by regulating the Wnt and VEGF signaling pathways. Specifically, circ_009789 and circ_004229 identified in this model could target miR-298-5p to regulate the PKC signaling pathway, neuronal apoptosis, and glycolipid metabolism, which are closely related to the development of POCD [29]. The enrichment of cirCeNET in abnormal oxygen metabolism pathways leads to cognitive function and behavioral alterations by promoting apoptosis, affecting synaptic function, and disrupting neuroplasticity [107]. Another transcriptomic analysis, also from hippocampal samples, identified a circRNA network (cirCeNET) associated with POCD, comprising three circRNAs, three miRNAs, and six mRNAs. Bioinformatics analysis and qRT-PCR identified circ_0001634/miR-490-5p/Rbm47, circ_0001634/miR-490-5p/ Sostdc1, circ_0001634/miR-7001-5p/Sostdc1, circ_0001345/miR-7001-5p/Sostdc1, and circ_0001493/miR-7001-5p/Sostdc1 as potential novel diagnostic biomarkers and therapeutic targets [108]. Similar to the lncRNA-regulated ceRNA mechanism, dexmedetomidine could also attenuate neuroinflammation and microglia activation by targeting the circ-Shank3/miR-140-3p/TLR4 axis, which is potentially another important mechanism by which it exerts its neuroprotective effects against POCD [109].

## 4 PROSPECTS OF THE ceRNA MECHANISM IN POCD

The vast majority of patients with POCD typically experience temporary cognitive decline. However, if left untreated, some individuals may develop permanent cognitive impairment or even progress to dementia. Previous studies have shown that patients with early onset of postoperative delirium have a 12-fold increased risk of developing dementia [110]. Therefore, it is crucial to identify new biomarkers that facilitate early diagnosis and effective monitoring of treatment and prognosis.

Historically, research in molecular neurobiology has primarily focused on protein-coding genes. However, they account for only a small portion of the entire transcriptome, while non-coding RNAs (ncRNAs) are increasingly recognized as important regulators of various cellular processes. As key components of epigenetic mechanisms, ncRNAs have been shown to regulate various cellular activities at multiple levels, including histone methylation, transcription, transcript splicing, translation, mRNA stability, and even the regulation of other ncRNAs. While current studies largely emphasize the role of ncRNAs in modulating gene expression, future research may uncover additional and diverse intracellular functions of these molecules. In a previous review, we summarized in detail the role of epigenetic mechanisms in POCD, including a section on the regulatory mechanisms of ncRNAs in POCD [111].

Notably, ncRNAs are particularly abundant in brain tissue, and their spatiotemporally differentiated expression pattern suggests that they are far from being transcriptional byproducts. Many ncRNAs have been shown to derive from genes encoding synaptic proteins or from genes responsible for neurodegenerative disorders. In some cases, the ceRNA networks established by these ncRNAs may be disease-specific. Interestingly, in POCD models, the same ncRNA can exert entirely different effects depending on the target miRNAs with which it interacts, highlighting the complexity and context-dependent nature of ceRNA-mediated regulation. These divergent findings underscore the need for comprehensive construction and characterization of ceRNA networks in various signaling contexts. Considering the nature of POCD, using a single ncRNA target as a biomarker is challenging and insufficient. Instead, integrating multiple molecular components, such as lncRNAs, circRNAs, pseudogenes, miRNAs, and mRNAs, into a ceRNA network may enhance diagnostic sensitivity and specificity. Furthermore, constructing ceRNA networks helps identify new molecular mechanisms of gene regulation, contributing to a better understanding of POCD pathogenesis and revealing new therapeutic targets.

Encouragingly, the ceRNA network holds clear advantages as a biomarker for the diagnosis and treatment of POCD, as ncRNAs are highly stable and readily detectable in circulation, making them ideal candidates for non-invasive applications. However, there are still many limitations that must be addressed in current research. Firstly, the ceRNA regulatory mechanism is not a simple point-to-point regulation, but a complex regulatory network. Our current understanding of the ceRNA regulatory mechanism remains rudimentary. In the future, applying new bioinformatics analysis methods and computational tools to construct a complex ceRNA network involving a great number of RNA molecules will be invaluable for predicting and discovering new biomarkers or potential therapeutic targets for diseases. Secondly, although ceRNA networks have opened up new avenues for molecular diagnosis and detection of POCD, only a small fraction of ceRNAs have been characterized at the molecular level. Given the complexity of these networks, their interactions must be validated by more experiments. Finally, ceRNA networks are potential therapeutic targets and biomarkers for personalized medicine. The emergence of RNA therapeutics offers a foundation for targeting ncRNAs in the prevention and treatment of POCD. Although the efficiency and specificity of delivery still need to be optimized, such minimally invasive drug delivery has brought new hope for treating many diseases. Considering the complex ceRNA network, more in-depth functional experiments are needed to fully elucidate the molecular functions of the ceRNA network in POCD and ultimately develop novel therapies.

## CONCLUSION

In this review, we focused on the classification and molecular signatures of ncRNAs, as well as their roles in ceRNA mechanisms in POCD (Table **1**, Figs. **1** and **2**). Considering the potential role of ncRNA-mediated ceRNA mechanisms in both therapeutic interventions and clinical diagnosis, we further evaluated their prospects as diagnostic biomarkers and as the basis for developing novel treatment strategies for POCD.

## Figures and Tables

**Table 1:** The identified ceRNA networks in POCD.

**ceRNA**	**Mechanism**	**Models**	**References**
LncRNAGAS5- miR-137-TCF4	Neuroinflammation regulation	Sevoflurane exposed mice	[67]
lncNONMMUT055714-miR-7684-5p-SORLA	Accumulation of Aβ and p-tau; neuroinflammation; oxidative stress; neuronal apoptosis	POCD mice with orthopedic surgery	[68, 73]
lncRNA Rian-miR-143-3p- LIMK1	Neuronal apoptosis	Sevoflurane exposed mice	[69]
lncRNA OIP5-AS1-miR-186-5p	Neuroinflammation; oxidative stress; neuron apoptosis	Isoflurane exposed mice	[70]
lncMEG3-miR-7-5p	Neuroinflammation; oxidative stress	Isoflurane exposed mice	[71]
lncMEG3-has-miR-106a-5p-SIRT3	Neuroinflammation; oxidative stress	POCD mice with orthopedic surgery	[72]
SNHG14/miR-340/NF-κB	Neuroinflammation	POCD mice with exploratory laparotomy	[74]
circAKT3-miR-106a-5p-HDAC4-MEF2C	Neuron apoptosis	POCD mice with orthopedic surgery	[75]
circUBE3B-miR-326/MYD88	Neuroinflammation; neuron apoptosis	Sevoflurane treated neurons	[76]
circ_0016760- miR-145	Neuroinflammation; oxidative stress	Sevoflurane exposed rats	[77]
circ_009789/circ_ 004229-miR-298-5P	PKC signaling pathway; neuron apoptosis; glycolipid metabolism pathway.	POCD mice with exploratory laparotomy	[18]

## LIST OF ABBREVIATIONS

AD: Alzheimer's Disease
ApoE4: Apolipoprotein E4
Aβ: Amyloid-β
ceRNA: Competing Endogenous RNA
circRNAs: Circular RNAs
CNS: Central Nervous System
eRNAs: Enhancer RNAs
HD: Huntington's Disease
lncRNAs: Long Non-coding RNAs
miRNAs: microRNAs
MREs: miRNA Response Elements
MS: Multiple Sclerosis
ncRNAs: Non-coding RNAs
PD: Parkinson's Disease
piRNAs: Piwi-interacting RNAs
PND: Postoperative Neurocognitive Deficit
POCD: Postoperative Cognitive Dysfunction
RISCs: RNA-induced Silencing Complexes
TBI: Traumatic Brain Injury
UTR: Untranslated Region
